# Direct medical costs of respiratory infections in adults: A multicenter retrospective analysis in Thai Nguyen, Vietnam

**DOI:** 10.1371/journal.pone.0354461

**Published:** 2026-07-23

**Authors:** Ba Khuong Cao, Thi Phuong Lan Nguyen, Maarten J. Postma, Phuong Sinh Nguyen, Chi Kien Duong, Jurjen van der Schans

**Affiliations:** 1 Department of Health Sciences, University of Groningen, University Medical Center Groningen, Groningen, Netherlands; 2 Faculty of Public Health, Thai Nguyen University of Medicine and Pharmacy, Thai Nguyen, Vietnam; 3 Centre of Excellence in Higher Education for Pharmaceutical Care Innovation, Universitas Padjadjaran, Bandung, Indonesia; 4 Department of Economics, Econometrics, and Finance, University of Groningen, Groningen, Netherlands; 5 Division of Pharmacology and Therapy, Faculty of Medicine, Universitas Airlangga, Surabaya, Indonesia; 6 Department of Rehabilitation, Thai Nguyen University of Medicine and Pharmacy, Thai Nguyen, Vietnam; 7 Department of Rehabilitation, Thai Nguyen National Hospital, Thai Nguyen, Vietnam; 8 Phu Binh General Hospital, Thai Nguyen, Vietnam; 9 Department of Economics, Econometrics and Finance, Faculty of Economics and Business, University of Groningen, Groningen, Netherlands; 10 Faculty of Management Sciences, Open University, Heerlen, Netherlands; Menzies School of Health Research: Charles Darwin University, AUSTRALIA

## Abstract

**Background:**

Respiratory infections remain a significant health and economic burden in low- and middle-income countries, including Vietnam. This study aimed to estimate the direct medical costs of respiratory infections among adults in Thai Nguyen, Vietnam and identify some influencing factors.

**Methods:**

In this retrospective study, we analyzed direct medical costs of inpatient aged ≥18 discharged with respiratory infections (ICD-10 codes: J00 – J22) in seven hospitals in Thai Nguyen, Vietnam. Records of patients admitted between 11 November 2022 and 30 June 2025 were included. A generalized gamma linear mixed model with log-link function was used to identify influencing factors, including patient demographics and infection-related characteristics. All costs are expressed in US$, adjusted to 2025 levels.

**Results:**

The analysis included 25,081 inpatients with 28,016 respiratory infection episodes. Most were female (54.3%), Kinh ethnicity (70.0%), and aged ≥60 (58.1%). Pneumonia (J18) was the most common primary diagnosis (58.5%), followed by bacterial pneumonia (J15, 18.7%). Common secondary diagnoses included hypertension (27.1%), gastro-oesophageal reflux disease (7.8%), and type 2 diabetes (6.9%). The median total medical cost per inpatient episode was US$174.9 (IQR 104.7–243.3), mainly driven by hospital bed days (median US$83.5), followed by medicine costs (US$52.7), and laboratory tests (US$12.7). Pneumonia was the most expensive disease, with median costs of US$218.8 (J15), US$1,179.4 (J16), and US$181.5 (J18). Primary diagnosis, age, gender, hospital level, length of stay, and additional secondary diagnosis were associated with higher direct medical costs.

**Conclusion:**

Hospitalized adults with respiratory infections incurred a median direct medical cost of US$174.9 in Thai Nguyen, Vietnam. Costs were substantially higher for lower respiratory tract infections and were influenced by hospital level, length of stay, and comorbidities. These findings highlight the need for preventive measures and efficient hospital resource allocation in Vietnam.

## Introduction

Respiratory infections remain a significant global health burden, particularly in low- and middle-income countries (LMICs) [1,2]. The 2021 global incidence of upper respiratory infections was estimated at 166,770.73 per 100,000 population, while that of lower respiratory infections was 4,283.61 per 100,000 population [2]. In the same year, an estimated 344 million cases of lower respiratory infections and 2.18 million associated deaths were recorded worldwide [3]. LMICs accounted for the majority of cases (83.8%) and deaths (75.2%). The burden was higher among individuals aged ≥50 years, which represented 54.0% of cases and 67.8% of deaths globally [3]. In Vietnam, a middle-income country, respiratory infections continue to pose a substantial health challenge [4,5], especially as the population is aging, with individuals aged ≥65 years expected to comprise 21.5% of the total population by 2069 [6].

Despite the high burden of respiratory infections in Vietnam, there is limited evidence on their direct medical costs across hospital levels. Health agencies and policymakers face the challenge of insufficient cost information when estimating the burden of diseases, allocating resources, and developing effective health insurance policies [7]. A better understanding of disease management costs is essential for guiding health agencies in making optimal resource allocations [8]. Such data are also crucial for designing sustainable health insurance policies and determining appropriate reimbursement rates [9]. In addition, they represent key inputs in economic evaluations of interventions, which serve as effective tools for identifying the most beneficial options within limited budgets [10,11]. However, due to the scarcity of local cost data, economic evaluations in Vietnam often rely on cost parameters derived from studies conducted in other settings [12,13]. This reliance reduces the accuracy and relevance of the evaluations, as significant disparities exist between countries in aspects such as healthcare system organization, living standards, local resource prices, and treatment procedures.

Therefore, this study was conducted to provide policymakers and researchers with scientific evidence to guide resource allocation, policy development, and further studies on cost-effectiveness evaluation. The objective of this study was to estimate the direct medical costs of respiratory infections among adults in Thai Nguyen, Vietnam and to identify factors influencing the costs.

## Methods

A retrospective observational study was conducted to assess the direct medical costs of adult inpatients who were diagnosed at discharge with respiratory infections, as classified by the International Classification of Disease (ICD) – 10 codes.

### Study population and settings

Patient records were retrieved for individuals who met the following inclusion criteria: (1) Inpatients aged 18 years and above at the time of admission; (2) Discharge diagnosis classified as: J00-J06 (Acute upper respiratory infections), J09-J18 (Influenza and pneumonia), or J20-J22 (Other acute lower respiratory infections). Records of all patients admitted between 11 November 2022 and 30 June 2025 were included to avoid the pandemic period in Vietnam, when treatment activities had returned to normal, and to increase the sample size. The specific extraction period for the datasets depended on the hospital’s current available software, storage capacity, and coding practices. Access to anonymized datasets was granted by the hospitals from 7 October to 27 October 2025. The specific extraction periods and access dates for all hospitals are provided in the S1 Table in the Supporting Information.

At the time of this study, Vietnam had a four-tiered healthcare system comprising national, provincial, district (regional), and commune-level facilities. Individuals were registered at a primary healthcare facility based on their permanent residential address. Inpatients diagnosed with the included diseases were treated at district level (regional) or higher-level facilities. Medical costs were recorded in hospital management information systems. Individual hospitals used different software systems, which were generally not interconnected across institutions [14]. Patient data were subsequently reported to the health insurance management database for reimbursement processing. The health insurance reimbursement rates for medical services and treatments varied according to patients’ insurance category, type of service provided, compliance with referral regulations, and whether the services fall within the approved benefit package and within the regulated pricing framework. Reimbursement rates generally ranged from 80% to 100% of eligible costs. However, reimbursement was not applied or was significantly reduced for services outside the approved package, for charges exceeding regulated prices, or when patients bypass the referral system, particularly when seeking care at higher-level hospitals.

**Fig 1:**
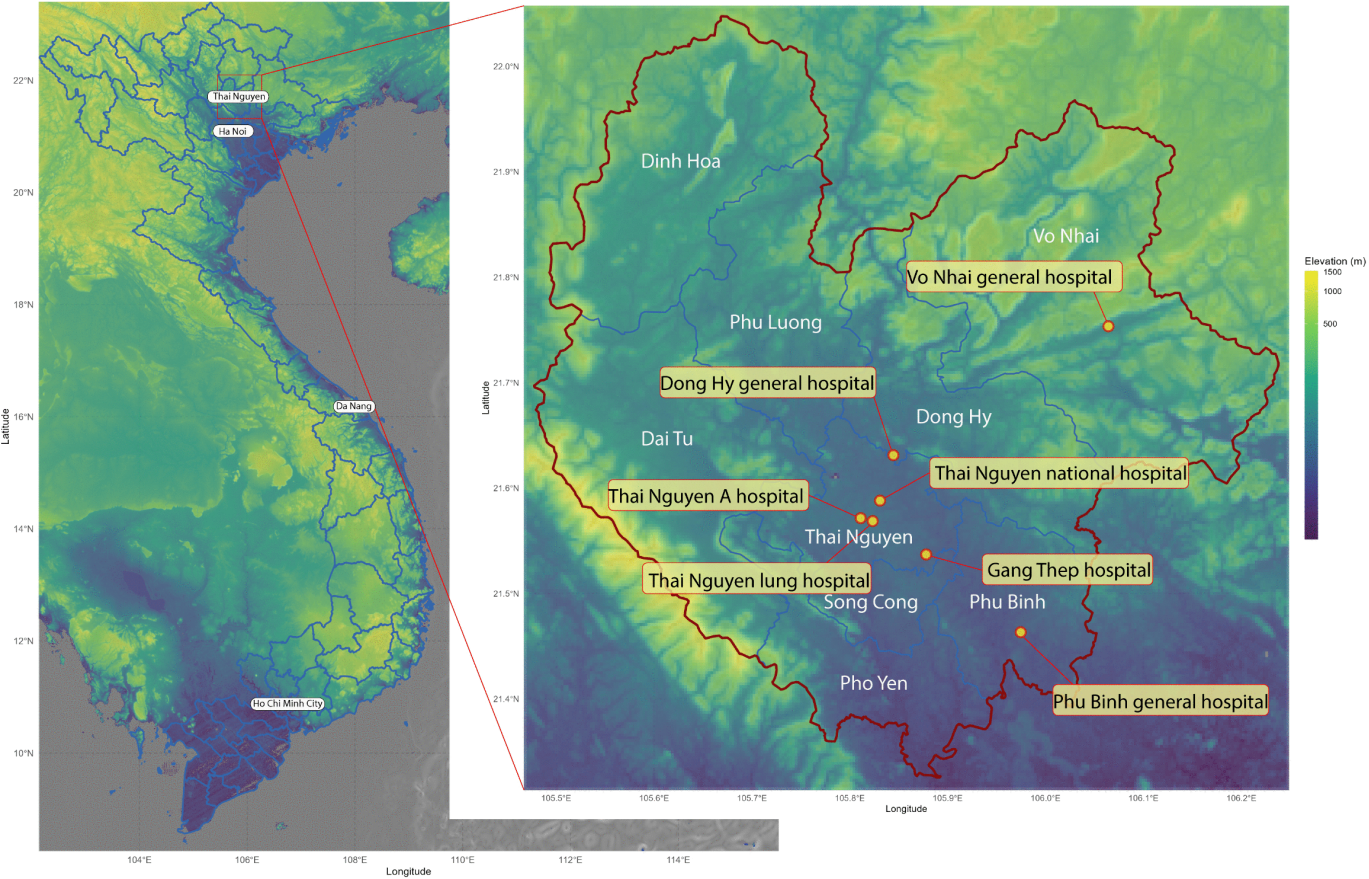
Geographic distribution of the selected hospitals in Thai Nguyen province, Vietnam. The map displays the locations of the seven selected hospitals in Thai Nguyen Province, overlaid on elevation data. Administrative boundaries correspond to the administrative system prior to the 2025 restructuring of Vietnam’s administrative units. The maps were created in R using open-source administrative boundary data from GADM version 4.1 (https://gadm.org/license.html) and elevation data from the U.S. Geological Survey (USGS) Shuttle Radar Topography Mission (https://www.usgs.gov/centers/eros/science/usgs-eros-archive-digital-elevation-shuttle-radar-topography-mission-srtm).

This study was conducted in Thai Nguyen province, Vietnam, a regional center in the northeastern part of the country. About 80 km north of Hanoi, Vietnam’s capital, the province connects the capital region with the northern midland and mountainous areas. Thai Nguyen features a diverse landscape of mountains, hills, valleys, and delta plains, leading to variations in accessibility, infrastructure, and economic activities across urban and rural areas. This geographic and economic diversity may affect patterns of healthcare use and the economic burden of illness. Seven hospitals were selected to represent the organizational structure of the Vietnamese healthcare system and the province’s geographic diversity. The selected hospitals included one national hospital (Thai Nguyen national hospital), three provincial hospitals (Gang Thep hospital, Thai Nguyen lung hospital, Thai Nguyen A hospital), and three regional hospitals (Phu Binh general hospital in the delta area, Dong Hy general hospital in the highland area, Vo Nhai general hospital in the mountainous area) (**Fig 1**). By including hospitals across all major tiers and diverse geographic areas, these sites reasonably reflect the variation in hospital services and patient populations in Vietnam, particularly in provinces with similar socioeconomic characteristics.

### Data collection and cleaning

Claim data from hospitals were accessed to provide estimates of direct medical costs of respiratory infections [15]. Data covering the entire hospitalization period for each patient were obtained from medical records. These records included: (1) general characteristics of patients including age, gender, ethnicity, occupation; (2) respiratory infection information including primary and secondary diagnosis (ICD-10 codes), dates of admission and discharge, and hospital name; (3) all healthcare services received during the hospitalization, along with the corresponding units, quantities, costs, cost categories, and amounts paid by insurance and by patients.

Due to the use of different software and coding systems for data management, the provided datasets differed significantly in terms of variable types, formats, and names. Variables were standardized in both format and type before being merged into a single long format dataset containing all health services received by each patient. This dataset was subsequently transformed into wide format using key variables such as patient code, date of admission, and hospital of treatment. Appropriate descriptive statistics were performed to detect outliers, which could indicate potential data entry errors. Modifications were made, when necessary, based on consultations and verification by hospital data management staff.

### Statistical analysis

The total hospitalization cost was calculated as the sum of all service costs, representing the total bill charges per admission. Costs were further disaggregated into the following categories: laboratory tests, imaging diagnostics, medicines, blood products, procedures, medical supplies, consultations, hospital bed days, transportation, and other services.

To more accurately reflect changes in local resource prices, all costs were adjusted for inflation using the local currency’s inflation rate, based on the Gross Domestic Product (GDP) implicit price deflators [16]. The adjusted values were calculated by multiplying the original amount by the ratio of the GDP deflator for 2025 (numerator) to that of the original year (denominator). The year of admission was considered the original year. To facilitate international comparisons, the adjusted costs were converted to US dollars (US$) using the exchange rate of 2025, which was 26,312 VND equal to 1 US$. GDP implicit price deflators were sourced from the International Monetary Fund’s World Economic Outlook [17].

Because cost data are typically right-skewed, costs were summarized using median and interquartile range (IQR). To provide additional details that may be useful for purposes such as economic evaluations, the mean and standard deviation were also reported.

A generalized linear model (GLM) was used to identify factors influencing costs. The Modified Park’s Test was applied to assess the appropriate distribution family. The test was conducted by first fitting an initial GLM, specified with a Gaussian distribution and log link, including only fixed effects. Pearson residuals were then obtained from this model and squared. The logarithm of the squared residuals was regressed on the logarithm of the fitted values. The estimated coefficient from this auxiliary regression was used to infer the appropriate variance function, where a coefficient near 0 indicates a Gaussian distribution, a value near 1 corresponds to a Poisson distribution, and a value near 2 suggests a Gamma distribution [18]. The estimated coefficient of 1.29 indicates a mean-variance relationship intermediate between Poisson and Gamma specifications. This supports the use of a Gamma distribution for modelling positively skewed cost data in this study. Consistent with recommendations from a previous study [19], a Gamma distribution with a log-link function was ultimately used. To account for the potential clustering of patients due to the possibility of one patient having more than one hospitalization or being treated at the same hospitals, a generalized linear mixed model was adopted. Independent variables included gender, age, occupation, primary diagnosis (ICD-10 code), secondary diagnosis (ICD-10 code), and length of stay. Only observations with complete data across all variables were included in the univariate and multivariable GLM analyses. ICD-10 codes with fewer than 10 recorded episodes were aggregated into an “Other” category in the cost analysis to improve the reliability of cost estimates. Data cleaning and all statistical analyses were performed using R.

### Ethical considerations

This study analyzed de-identified data, where personal information such as patient codes were encrypted to ensure anonymity [20]. The authors had no access to identifying information for individual participants at any stage of the study. Ethical approval was obtained from the Ethics Committee of Thai Nguyen University of Medicine and Pharmacy. This study was conducted with the permission of participating hospitals.

## Results

The general characteristics of the patients and their respiratory infection episodes are summarized in **Table 1**. A total of 25,081 patients with 28,016 episodes, who were admitted between 11 November 2022, and 30 June 2025, were included in the analysis. Among these patients, 54.3% were female and 70.0% were Kinh ethnicity. More than half were aged 60 years or older (58.1%) and were farmers (56.3%).

**Table 1:** General characteristics of the patients and episodes of respiratory infections.

Characteristic	n(%)
**Patients (n = 25,081)**	
**Gender**	
Female	12,560 (54.3)
Male	10,592 (45.7)
Unknown^†^	1,929 (7.7)
**Ethinicity**	
Kinh	15,228 (70.0)
Minority	6,541 (30.0)
Unknown^†^	3,312 (13.2)
**Age group**	
18–29	1,493 (6.0)
30–44	3,461 (13.8)
45–59	5,558 (22.2)
60 and older	14,569 (58.1)
**Occupation**	
Farmer	12,052 (56.3)
Worker	2,958 (13.8)
Retired	3,231 (15.1)
Unemployed	880 (4.1)
Governmental/Office staff/Professionals	866 (4.0)
Freelance	589 (2.8)
Students	269 (1.3)
Others	570 (2.7)
Unknown^†^	3,666 (14.6)
**Episodes (n = 28,016)**	
**Partially covered by insurance**	
Yes	27,139 (96.9)
No	877 (3.1)
**Hospital level**	
Regional	7,617 (27.2)
Provincial	16,880 (60.3)
National	3,519 (12.6)
**Primary diagnosis at discharge (ICD-10 code)**	
J00: Acute nasopharyngitis [common cold]	65 (0.2)
J01: Acute sinusitis	805 (2.9)
J02: Acute pharyngitis	1,452 (5.2)
J03: Acute tonsillitis	250 (0.9)
J04: Acute laryngitis and tracheitis	302 (1.1)
J05: Acute obstructive laryngitis [croup] and epiglottitis	4 (0.0)
J06: Acute upper respiratory infections of multiple and unspecified sites	60 (0.2)
J09: Influenza due to certain identified influenza virus	553 (2.0)
J10: Influenza due to identified seasonal influenza virus	64 (0.2)
J11: Influenza, virus not identified	187 (0.7)
J12: Viral pneumonia, not elsewhere classified	8 (0.0)
J13: Pneumonia due to Streptococcus pneumoniae	3 (0.0)
J15: Bacterial pneumonia, not elsewhere classified	5,227 (18.7)
J16: Pneumonia due to other infectious organisms, not elsewhere classified	51 (0.2)
J17: Pneumonia in diseases classified elsewhere	9 (0.0)
J18: Pneumonia, organism unspecified	16,387 (58.5)
J20: Acute bronchitis	2,285 (8.2)
J22: Unspecified acute lower respiratory infection	304 (1.1)
**Number of secondary diagnosis (ICD-code)**	
0	5,599 (20.0)
1	11,357 (40.5)
2	5,210 (18.6)
3	2,809 (10.0)
≥ 4	3,041 (10.9)
**Most frequent secondary diagnosis (ICD-10 code)**	
I10: Essential (primary) hypertension	7,602 (27.1)
K21: Gastro- oesophageal reflux disease	2,186 (7.8)
E11: Type 2 diabetes mellitus	1,925 (6.9)
H81: Disorders of vestibular function	1,392 (5.0)
I20: Angina pectoris	1,205 (4.3)
J45: Asthma	1,130 (4.0)
I50: Heart failure	1,016 (3.6)
J02: Acute pharyngitis	977 (3.5)

† The percentage was calculated based the total number of patients/episodes.

The most frequent primary diagnosis was J18-Pneumonia, organism unspecified (58.5%), followed by J15-Bacterial pneumonia, not elsewhere classified (18.7%), and J20-Acute bronchitis (8.2%). The most common secondary diagnoses were I10-Essential (primary) hypertension (27.1%), K21-Gastro-oesophageal reflux disease (7.8%), E11-Type 2 diabetes mellitus (6.9%). The episodes included in the analysis originated from provincial hospitals (60.3%), regional hospitals (27.2%) and the national hospital (12.6%). Most of the episodes (96.9%) were partially covered by health insurance.

**Fig 2:**
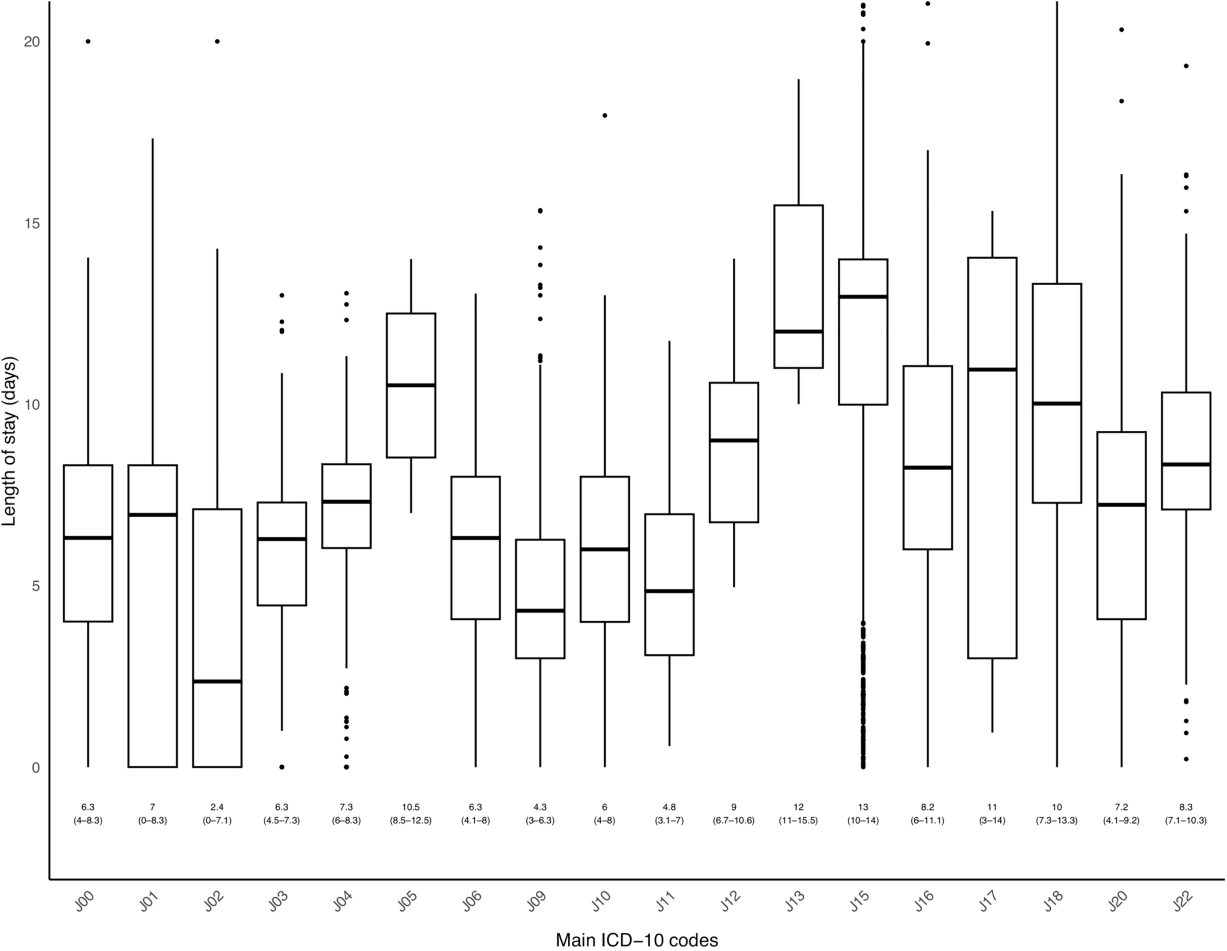
Length of stay of episodes by primary diagnosis (ICD-10 code). Values are presented as median (interquartile range, IQR) in the plot; J00: Acute nasopharyngitis [common cold]; J01: Acute sinusitis; J02: Acute pharyngitis; J03: Acute tonsillitis; J04: Acute laryngitis and tracheitis; J05: Acute obstructive laryngitis [croup] and epiglottitis; J06: Acute upper respiratory infections of multiple and unspecified sites; J09: Influenza due to certain identified influenza virus; J10: Influenza due to identified seasonal influenza virus; J11: Influenza, virus not identified; J12: Viral pneumonia, not elsewhere classified; J13: Pneumonia due to Streptococcus pneumoniae; J15: Bacterial pneumonia, not elsewhere classified; J16: Pneumonia due to other infectious organisms, not elsewhere classified; J17: Pneumonia in diseases classified elsewhere; J18: Pneumonia, organism unspecified; J20: Acute bronchitis; J22: Unspecified acute lower respiratory infection.

The length of stay among inpatients with respiratory infections is illustrated in **Fig 2**. Overall, the median length of stay was 9.3 days (IQR 6.8–13.0). For upper respiratory infections, the median durations were 6.3 days (IQR 4.0–8.3) for J00-Acute nasopharyngitis [common cold], 7.0 days (IQR 0.0–8.3) for J01-Acute sinusitis, 2.4 days (IQR 0.0–7.1) for J02-Acute pharyngitis, 6.3 days (IQR 4.4–7.3) for J03-Acute tonsillitis, 7.3 days (IQR 6.0–8.3) for J04-Acute laryngitis and tracheitis, and 6.3 days (IQR 4.0–8.0) for J06-Acute upper respiratory infections of multiple and unspecified sites.

Among influenza cases, the median lengths of stay were 4.3 days (IQR 3.0–6.3) for J09-Influenza due to certain identified influenza viruses, 6.0 days (IQR 4.0–8.0) for J10-Influenza due to other identified influenza virus, and 4.8 days (IQR 3.1–7.0) for J11-Influenza, virus not identified.

For pneumonia cases, the median lengths of stay were 13.0 days (IQR 10.0–14.0) for J15-Bacterial pneumonia, not elsewhere classified, 8.3 days (IQR 6.0–11.1) for J16-Pneumonia due to other infectious organisms, not elsewhere classified, and 10.0 days (IQR 7.3–13.3) for J18-Pneumonia, organism unspecified. The median length of stay was 7.2 days (IQR 4.1–9.2) for J20-Acute bronchitis and 8.3 days (IQR 7.1–10.3) for J22-Unspecified acute lower respiratory infection. Additional details are provided in S2 Table in the Supporting Information.

**Fig 3:**
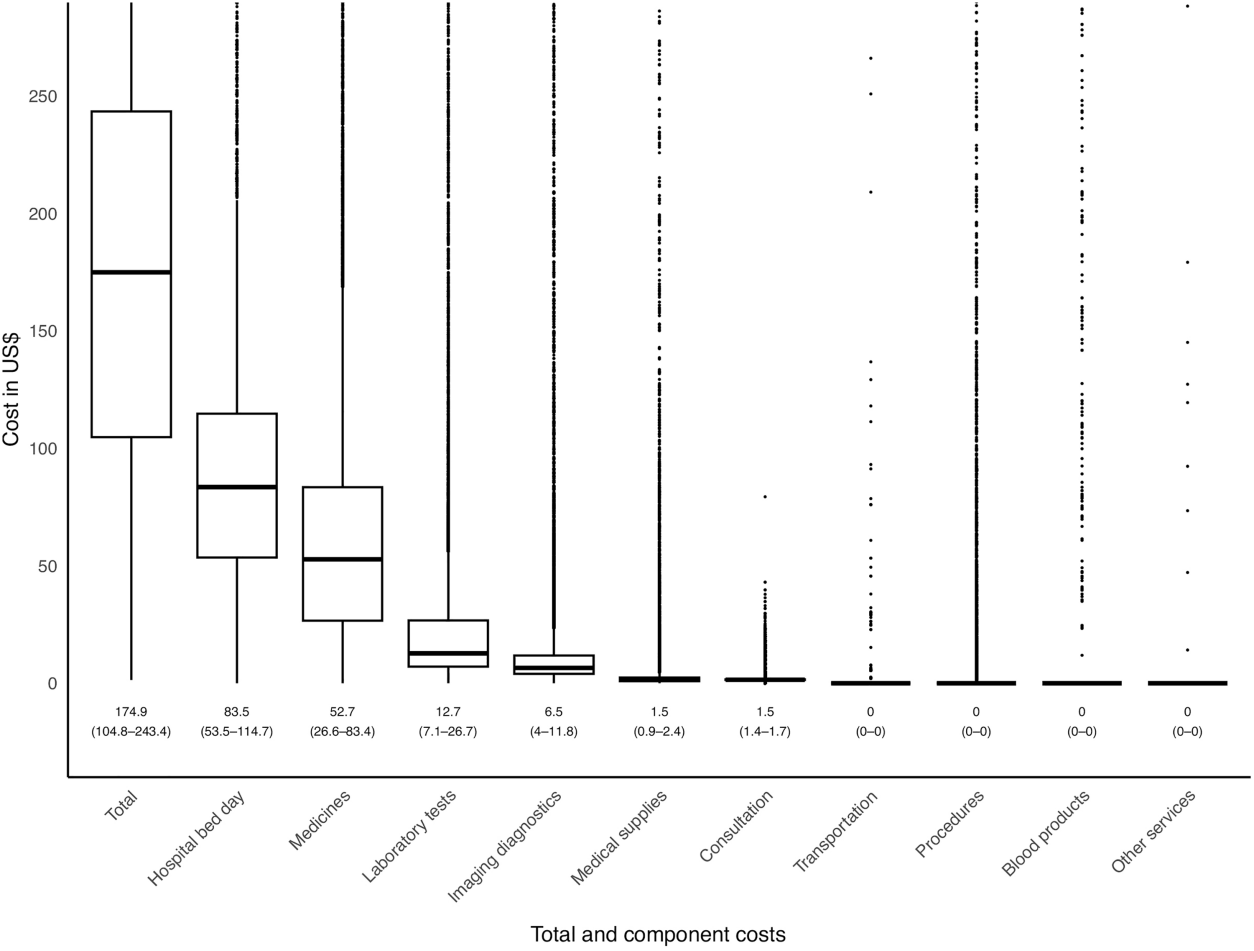
The direct medical costs of inpatients diagnosed with respiratory infections. Values are presented as median (interquartile range, IQR) in the plot; The transportation cost is typically for inpatients who need to be referred to another facility, require emergency transfer, or use medical transportation services designated by the hospital.

The direct medical costs of inpatients diagnosed with respiratory infections are summarized in **Fig 3**. Overall, the median total medical cost was US$174.9 (IQR 104.7–243.3), of which US$153.9 (IQR 93.5–214.0) was covered by health insurance. Among the cost components, the majority of expenses were attributed to hospital bed days (median US$83.5, IQR 53.5–114.7), followed by medicines (median US$52.7, IQR 26.6–83.4), laboratory tests (median US$12.7, IQR 7.1–26.7), and imaging diagnostics (median US$6.5, IQR 4.0–11.8).

The median direct medical costs varied substantially across disease categories (**Table 2**). For upper respiratory tract infections, J00-Acute nasopharyngitis [common cold] had a median cost of US$121.3 (IQR 77.0–176.5), while J01-Acute sinusitis followed closely at US$112.5 (IQR 28.6–160.1). J02-Acute pharyngitis was less costly, with a median of US$45.0 (IQR 8.4–110.3), whereas J03 – Acute tonsillitis and J04-Acute laryngitis and tracheitis had median costs of US$92.9 (IQR 66.0–131.5) and US$120.4 (IQR 68.5–150.9), respectively. The highest cost in this group was for J06-Acute upper respiratory infections of multiple and unspecified sites, with a median of US$140.7 (IQR 77.5–203.8).

**Table 2:** Direct medical costs of episodes by primary diagnosis (ICD-10 code) in US$.

Variable	J00 *Acute nasopharyngitis [common cold]*	J01 *Acute sinusitis*	J02 *Acute pharyngitis*	J03 *Acute tonsillitis*	J04 *Acute laryngitis and tracheitis*	J06 *Acute upper respiratory infections of multiple and unspecified sites*	J09 *Influenza due to certain identified influenza virus*	J10 *Influenza due to identified seasonal influenza virus*	J11 *Influenza, virus not identified*	J15 *Bacterial pneumonia, not elsewhere classified*	J16 *Pneumonia due to other infectious organisms, not elsewhere classified*	J18 *Pneumonia, organism unspecified*	J20 *Acute bronchitis*	J22 *Unspecified acute lower respiratory infection*	Others*
	n = 65	n = 805	n = 1,452	n = 250	n = 302	n = 60	n = 553	n = 64	n = 187	n = 5,227	n = 51	n = 16,387	n = 2,285	n = 304	n = 24
						**Total costs – Median (Interquartile range – IQR)**									
Total amount	121.3 (77.0–176.5)	112.5 (28.6–160.1)	45.0 (8.4–110.3)	92.9 (66.0–131.5)	120.4 (68.5–150.9)	140.7 (77.5–203.8)	94.0 (59.2–196.1)	758.4 (261.0–1,205.1)	81.3 (53.8–125.0)	218.8 (180.1–269.8)	1,179.4 (248.7–3,390.7)	181.5 (113.5–247.3)	111.8 (71.9–161.8)	169.2 (124.8–239.9)	251.3 (146.2–345.3)
Covered by insurance	87.7 (55.3–150.5)	92.3 (0.0–136.2)	34.1 (5.1–98.6)	81.6 (56.6–119.0)	104.2 (56.7–136.7)	118.7 (57.4–182.1)	78.2 (46.8–151.4)	559.5 (213.8–990.2)	67.1 (42.9–103.9)	192.1 (151.7–235.2)	1,084.7 (215.8–2,611.8)	162.0 (103.9–219.7)	98.6 (56.4–146.1)	145.8 (107.6–203.7)	166.4 (117.0–317.0)
Paid by patients	16.1 (6.2–35.2)	11.8 (2.3–28.7)	3.9 (0.0–13.5)	9.3 (0.0–22.7)	8.0 (0.0–24.4)	13.6 (2.5–35.8)	14.1 (5.1–35.7)	73.8 (16.7–168.5)	13.0 (3.7–32.0)	22.1 (3.4–48.9)	70.5 (20.3–479.3)	7.3 (0.0–32.1)	11.6 (0.0–28.1)	18.4 (6.0–48.9)	37.6 (5.9–115.0)
						**Cost components – Median (Interquartile range – IQR)**									
Laboratory tests	6.4 (5.3–12.3)	5.3 (0.0–7.6)	1.9 (0.0–6.9)	5.9 (1.9–6.3)	4.7 (4.4–6.1)	7.6 (6.1–15.3)	10.9 (8.4–31.9)	97.1 (34.4–179.9)	10.8 (8.6–19.3)	26.0 (14.3–42.2)	153.1 (29.0–470.9)	12.6 (7.3–24.9)	8.6 (6.0–17.2)	14.6 (9.5–23.0)	21.1 (9.9–4.1)
Imaging diagnostics	2.7 (0.0–6.0)	3.9 (0.0–8.0)	2.7 (0.0–5.4)	0.0 (0.0–2.7)	0.0 (0.0–2.7)	4.2 (2.7–6.6)	5.0 (4.0–9.6)	24.0 (7.1–73.0)	6.0 (4.6–10.0)	9.3 (6.0–18.0)	36.1 (12.1–170.2)	6.7 (4.0–11.2)	4.1 (2.7–8.5)	6.5 (4.2–12.0)	6.9 (4.6–25.1)
Medicines	41.0 (23.9–72.9)	41.5 (0.0–69.0)	11.4 (3.0–43.8)	40.1 (22.5–68.8)	45.5 (21.5–64.6)	41.1 (10.8–99.1)	8.6 (4.1–32.6)	211.8 (25.5–484.6)	8.6 (4.1–18.7)	62.1 (45.9–94.2)	406.8 (72.1–1,005.2)	55.4 (31.0–88.5)	34.6 (13.0–63.2)	53.8 (22.2–86.0)	78.9 (39.8–123.4)
Blood products	0.0 (0.0–0.0)	0.0 (0.0–0.0)	0.0 (0.0–0.0)	0.0 (0.0–0.0)	0.0 (0.0–0.0)	0.0 (0.0–0.0)	0.0 (0.0–0.0)	0.0 (0.0–0.0)	0.0 (0.0–0.0)	0.0 (0.0–0.0)	0.0 (0.0–0.0)	0.0 (0.0–0.0)	0.0 (0.0–0.0)	0.0 (0.0–0.0)	0.0 (0.0–0.0)
Procedures	0.0 (0.0–0.0)	0.0 (0.0–4.2)	0.0 (0.0–0.0)	0.0 (0.0–0.0)	0.0 (0.0–0.0)	0.0 (0.0–4.5)	0.0 (0.0–0.0)	0.0 (0.0–0.0)	0.0 (0.0–0.0)	0.0 (0.0–7.5)	28.9 (0.0–236.7)	0.0 (0.0–0.0)	0.0 (0.0–0.0)	0.0 (0.0–0.0)	0.0 (0.0–30.5)
Medical supplies	1.0 (0.7–2.6)	0.9 (0.0–1.6)	0.3 (0.0–1.0)	0.8 (0.5–1.3)	0.9 (0.6–1.2)	2.3 (0.7–3.1)	1.2 (0.7–3.3)	15.0 (6.4–28.6)	0.9 (0.5–1.4)	2.1 (1.3–2.8)	21.6 (4.0–91.3)	1.6 (0.9–2.4)	1.1 (0.4–2.0)	2.0 (1.1–3.1)	2.7 (1.6–6.6)
Consultation	1.5 (1.4–1.7)	1.5 (0.0–1.7)	1.5 (0.0–1.7)	1.4 (0.0–1.7)	1.7 (0.0–1.7)	1.7 (1.4–3.3)	1.7 (1.5–3.2)	6.6 (3.3–11.0)	1.5 (1.4–1.7)	1.5 (1.4–1.5)	4.0 (1.5–9.9)	1.5 (1.4–1.7)	1.4 (1.3–1.7)	1.5 (1.5–2.9)	1.7 (1.5–1.7)
Hospital bed day	50.7 (35.9–84.5)	50.2 (0.0–71.9)	20.2 (0.0–52.3)	42.7 (30.5–59.1)	62.4 (37.3–72.8)	72.4 (39.2–87.4)	60.2 (34.8–100.2)	318.4 (128.7–481.7)	48.9 (25.1–70.2)	107.1 (84.1–116.9)	388.4 (91.9–873.8)	90.2 (58.5–116.9)	53.5 (32.7–70.2)	78.2 (58.9–107.5)	105.3 (81.9–174.9)
Transportation	0.0 (0.0–0.0)	0.0 (0.0–0.0)	0.0 (0.0–0.0)	0.0 (0.0–0.0)	0.0 (0.0–0.0)	0.0 (0.0–0.0)	0.0 (0.0–0.0)	0.0 (0.0–0.0)	0.0 (0.0–0.0)	0.0 (0.0–0.0)	0.0 (0.0–0.0)	0.0 (0.0–0.0)	0.0 (0.0–0.0)	0.0 (0.0–0.0)	0.0 (0.0–0.0)
Other services	0.0 (0.0–0.0)	0.0 (0.0–0.0)	0.0 (0.0–0.0)	0.0 (0.0–0.0)	0.0 (0.0–0.0)	0.0 (0.0–0.0)	0.0 (0.0–0.0)	0.0 (0.0–0.0)	0.0 (0.0–0.0)	0.0 (0.0–0.0)	0.0 (0.0–0.0)	0.0 (0.0–0.0)	0.0 (0.0–0.0)	0.0 (0.0–0.0)	0.0 (0.0–0.0)

* Others include J05: Acute obstructive laryngitis [croup] and epiglottitis, J12: Viral pneumonia, not elsewhere classified, J13: Pneumonia due to Streptococcus pneumoniae, J17: Pneumonia in diseases classified elsewhere.

Among influenza cases, J09-Influenza due to certain identified influenza viruses had a median cost of US$94.0 (IQR 59.2–196.1), while J11-Influenza, virus not identified had a slightly lower median cost of US$81.3 (IQR 53.8–125.0). The highest cost among this influenza group was for J10-Influenza due to other identified influenza virus, at US$758.4 (IQR 261.0–1,205.1).

Among pneumonia cases, the highest median cost was US$1,179.4 (IQR 248.7–3,390.7) for J16-Pneumonia due to other infectious organisms, not elsewhere classified. This was followed by J15-Bacterial pneumonia, not elsewhere classified, with a median cost of US$218.8 (IQR 180.1–269.8), and J18-Pneumonia, organism unspecified, with US$181.5 (IQR 113.5–247.3). J22-Unspecified acute lower respiratory infection had a slightly lower median cost of US$169.2 (IQR 124.8–239.9). The lowest cost within this group was for J20-Acute bronchitis, with a median of US$111.8 (IQR 71.9–161.8). Additional details are provided in S2 Table Supplemental Material.

**Table 3** presents the results from a generalized gamma mixed model, identifying factors associated with the direct medical costs of respiratory infections. Each additional year of patient age was associated with an increase in the medical cost (Exp(Beta) = 1.00, 95% CI 1.00–1.00). Male patients incurred approximately 7% higher costs than female patients (Exp(Beta) = 1.07, 95% CI 1.05–1.08). The length of stay significantly increased the total costs by 17% per additional day (Exp(Beta) = 1.17, 95% CI 1.16–1.17). Each additional secondary ICD-10 code diagnosis was associated with approximately 2% increase in costs (Exp(Beta) = 1.02, 95% CI 1.01–1.3).

**Table 3:** Factors influencing direct medical costs of respiratory infection episodes analysed by gamma generalized linear mixed model.

Variable	Univariable analysis			Multivariable analysis		
	Exp(Beta)	95% CI	p-value	Exp(Beta)	95% CI	p-value
**Age in years**	1.01	1.01–1.01	*<0.001*	1.00	1.00–1.00	<0.001
**Gender** (Ref. Female)						
Male	1.09	1.06–1.11	*<0.001*	1.07	1.05–1.08	<0.001
**Ethnicity** (Ref. Kinh)						
Minority	0.99	0.97–1.02	*0.6*	1.00	0.98–1.02	>0.9
**Principal diagnosis (ICD-10 code)** (Ref. J00: Acute nasopharyngitis [common cold])						
J01: Acute sinusitis	0.88	0.72–1.07	*0.2*	0.89	0.77–1.04	0.15
J02: Acute pharyngitis	0.52	0.43–0.63	*<0.001*	0.47	0.40–0.55	<0.001
J03: Acute tonsillitis	1.33	1.07–1.64	*0.009*	1.56	1.31–1.85	<0.001
J04: Acute laryngitis and tracheitis	1.33	1.08–1.64	*0.008*	1.38	1.16–1.63	<0.001
J06: Acute upper respiratory infections of multiple and unspecified sites	0.97	0.74–1.28	*0.8*	1.02	0.82–1.27	0.9
J09: Influenza due to certain identified influenza virus	1.06	0.87–1.30	*0.6*	1.57	1.34–1.84	<0.001
J10: Influenza due to identified seasonal influenza virus	2.96	2.25–3.89	*<0.001*	2.79	2.26–3.44	<0.001
J11: Influenza – virus not identified	0.89	0.71–1.10	*0.3*	1.26	1.06–1.49	0.008
J15: Bacterial pneumonia – not elsewhere classified	2.94	2.42–3.57	*<0.001*	2.19	1.88–2.54	<0.001
J16: Pneumonia due to other infectious organisms – not elsewhere classified	6.21	4.66–8.30	*<0.001*	3.75	3.01–4.69	<0.001
J18: Pneumonia – organism unspecified	2.82	2.32–3.42	*<0.001*	2.21	1.90–2.57	<0.001
J20: Acute bronchitis	1.53	1.26–1.86	*<0.001*	1.30	1.12–1.51	<0.001
J22: Unspecified acute lower respiratory infection	1.72	1.40–2.12	*<0.001*	1.40	1.19–1.65	<0.001
Others (J05, J12, J13, J17)	2.81	1.94–4.08	*<0.001*	1.91	1.44–2.55	<0.001
**Length of stay in days**	1.18	1.18–1.19	*<0.001*	1.17	1.16–1.17	<0.001
**Number of secondary diagnosis (ICD-10 code)**	1.07	1.06–1.08	*<0.001*	1.02	1.01–1.03	<0.001
**I10: Essential (primary) hypertension** (Ref. No or only others)						
Yes (I10 or I10 with others)	1.19	1.17–1.22	<0.001	0.95	0.93–0.97	<0.001
**K21: Gastro-oesophageal reflux disease** (Ref. No or only others)						
Yes (K21 or K21 with others)	0.80	0.77–0.83	<0.001	0.83	0.81–0.86	<0.001
**E11: Type 2 diabetes mellitus** (Ref. No or only others)						
Yes (E11 or E11 with others)	1.32	1.27–1.38	<0.001	1.13	1.10–1.17	<0.001
**I20: Angina pectoris** (Ref. No or only others)						
Yes (I20 or I20 with others)	1.04	0.99–1.10	0.1	0.86	0.82–0.90	<0.001
**J45: Asthma** (Ref. No or only others)						
Yes (J45 or J45 with others)	1.13	1.08–1.19	<0.001	1.06	1.02–1.10	0.002
**I50: Heart failure** (Ref. No or only others)						
Yes (I50 or I50 with others)	1.33	1.27–1.41	<0.001	1.01	0.96–1.05	0.8
**J02: Acute pharyngitis** (Ref. No or only others)						
Yes (J02 or J02 with others)	0.76	0.72–0.80	<0.001	0.78	0.74–0.82	<0.001
**H81: Disorders of vestibular function** (Ref. No or only others)						
Yes (H81 or H81 with others)	1.04	0.99–1.08	0.14	0.93	0.90–0.97	<0.001

Ref.: Reference, Exp(Beta): Exponential of coefficient, CI: Confident interval.

Regarding the primary diagnosis, patients diagnosed with J16 – Pneumonia due to other infectious organisms, not elsewhere classified and J18 – Pneumonia, organism unspecified incurred costs that were approximately 3.76 times (95% CI 3.01–4.69) and 2.21 times (95% CI 1.90–2.57) higher, respectively, compared to those with J00 – Acute nasopharyngitis [common cold]. Similarly, patients with J10 – Influenza due to identified seasonal influenza virus had costs about 2.79 times higher (95% CI 2.26–3.44). In contrast, significantly lower costs were observed among patients with J02 – Acute pharyngitis (Exp(Beta) = 0.47, 95% CI 0.40–0.55) (**Table 3**).

## Discussion

This study studies the direct medical costs of inpatients aged 18 years and older who were diagnosed with respiratory infections as their primary disease in Thai Nguyen province, Vietnam. Additionally, this study identified influencing factors of the costs. The median direct medical cost was US$174.9 (IQR 104.7–243.3), associated with a median hospital stay of 9.3 days (IQR 6.8–13.0). Lower respiratory tract infections incurred substantially greater hospitalization costs than upper respiratory tract infections. For example, the median costs were approximately US$45.0 for J02 (Acute pharyngitis) and US$92.2 for J03 (Acute tonsillitis), whereas the median cost of the J16 (Pneumonia due to other infectious organisms, not elsewhere classified) reached US$1,179.4, and J15 (Bacterial pneumonia, not elsewhere classified) was US$218.8. Factors associated with higher costs included age, gender, primary diagnosis, length of stay, and the presence of secondary diagnoses.

In terms of absolute costs, the estimated median cost in this study was notably lower than those reported in some other studies. For instance, a meta-analysis reported significantly higher costs in high-income countries, with a mean of €1,780 in 2021 [21]. Another meta-analysis of studies in China reported a median direct medical cost of US$2,744 in 2021 [22]. In Southeast Asia, the mean cost per episode was €166.0 in 2021 [21]. These differences may reflect significant variation in healthcare system organization, living standards, local resource prices, treatment protocols, and the inclusion of elderly patients. Compared with a study conducted in Vietnam, the median cost in our study was considerably lower. That study reported a median direct medical cost of community-acquired pneumonia at US$465.0 in 2017 [23]. This higher figure could be explained by the inclusion of a more severe population (pneumonia hospitalized patients) and the setting in a tertiary hospital in Southern Vietnam.

The lower medical costs observed in this study should be interpreted in the context of Vietnam’s modest income levels and overall health expenditure of approximately 4.56% of GDP in 2023, which is far below the median of 8.12% in high-income countries [24]. These factors could shape both the healthcare pricing and the intensity of healthcare utilization. When comparing to the personal income, the estimated direct medical cost of US$174.9 is over 55% of an average monthly income, approximately US$315.4 in 2025 (8.3 million VND) [25]. Patients diagnosed with lower respiratory infections incurred significantly greater costs; for example, median costs for J16 and J10 were approximately 2.5 and 4.0 times higher than the Vietnamese monthly income, respectively. While a large proportion of the total medical costs was covered by health insurance, this places substantial pressure on health insurance funds, which are expected to face deficits [26]. On the other hand, the costs also place families under pressure, particularly in low-income households, while out-of-pocket payments remain significantly high in Vietnam [27], it was about 40% in 2022 [28] including direct non-medical costs (such as transportation, accommodation, and meals), indirect costs (such as productivity losses due to illness, including missed workdays of patients and family members) and a portion of direct medical costs.

Regarding the cost components, hospital bed days and medicines were the greatest contributors to the total direct medical costs, followed by imaging diagnostics and laboratory tests. This could be because respiratory infections are primarily treated as internal medical conditions, in which the treatments mainly involve pharmacotherapy and supportive care rather than invasive procedures or the use of blood products. These findings aligned with other studies, which also identified consumables, laboratory tests, and imaging as the major contributors [29]. Another study similarly reported that bed days and medications were the primary contributors to hospitalization costs [30].

Overall, the costs of lower respiratory tract infections were significantly higher than those of upper tract infections. This could be due to the greater severity levels of lower respiratory tract infections, which typically require longer hospital stays, more intensive use of healthcare resources. For example, patients diagnosed J15 - Bacterial pneumonia, not elsewhere classified stayed a median of 13.0 days, while it was only 7.0 days for J01-Acute sinusitis. Several studies highlighted the significant role of length of stay in driving the healthcare costs [31,32]. In routine clinical practice, more severe or complex cases are often referred to higher level hospitals, which can provide advanced diagnostic procedures, specialized treatments, and intensive care services, thereby increasing overall direct medical costs.

This study found that the cost for male patients was approximately 7.0% higher than for females. Studies showed that males are more likely to experience severe respiratory infections than females [33]. Several reported factors may contribute to this difference, such as lifestyle, health behaviour, anatomical differences, and hormonal influences [33]. In addition, the findings of this study align with other studies, which showed a significant increase in costs along with increasing patient age and the presence of comorbidities [21]. Older age is also a well-known factor for increasing the susceptibility and severity of several diseases, including respiratory infections due to impairments in the immune system and lung function [34,35].

Beyond patient-level and clinical factors, variation in direct medical costs should be interpreted within the broader context of Vietnam’s health financing and payment system. National health insurance plays a central role in financing inpatient care, covering approximately 80% of regulated direct medical costs [7]. At the system level, government-regulated price caps and tariff schedules standardize unit costs across services and aim to constrain total expenditures; however, aggregate costs are still primarily driven by service utilization intensity and prolonged hospitalization rather than unit price variation alone. Differences in costs across hospital tiers reflect both appropriate variation in case severity and structural features of the referral system, including higher resource intensity and the concentration of complex cases in tertiary hospitals. At the patient level, greater clinical severity and longer hospital stays increase healthcare resource use, and their financial impact is largely mediated through insurance reimbursement mechanisms that cover most inpatient expenditures. However, in cases where reimbursement from the Vietnam Social Security is restricted or not approved, hospitals may require patients to make out-of-pocket payments, thereby increasing the financial burden on households [7].

The findings of this study could provide important implications for health policy, disease management, and scientific research. They provide evidence to policymakers to estimate the overall burden of respiratory infections among adults and to guide budget allocations. From the perspective of the insurer, this evidence could support consideration of preventive interventions that may be economically beneficial and indirectly reduce treatment expenditures, particularly through interventions such as vaccinations, which have proven effective against respiratory infections. These findings can also serve as locally relevant input data for future economic evaluations of interventions aimed at reducing the burden of respiratory infections in the Vietnamese population. Indeed, a recent review of economic evaluations in Southeast Asia reported the need for local parameters when conducting new economic evaluations of vaccines [36].

This study has several strengths. The large number of all eligible patient records collected over a three-year period from hospitals across the major tiers of the healthcare system were included. This approach increased the sample size, which may improve statistical power for cost estimation and multivariable analyses. The seven hospitals were selected to reflect the diversity of hospital levels and geographic areas within the province, thereby helping to support the relevance and applicability of the study findings. However, the interpretation of the results should be considered in light of its limitations. First, although a national guideline exists for adopting ICD-10 coding, this study gathered data from multiple hospitals, so there might be some inconsistencies in actual practice regarding documentation quality, coder knowledge. However, this study analysed the diagnoses (ICD-10 codes) at discharge, which are likely more reliable as they were determined after evaluating examination results, the disease progression, and the patient’s response to treatment. Second, this study was unable to determine whether patients were transferred between hospitals in the analysis. Therefore, the direct medical costs may be underestimated due to limitations in data linkage, which made it impossible to capture all costs for patients transferred from other hospitals. Third, this study was unable to assess the influence of infection severity on direct medical costs, which is typically an important driver of medical expenses. Although severity could be partially reflected by the length of stay, it might still have affected the coefficients in the multivariable analysis. Fourth, this study analysed data from a single province in Vietnam. Although several hospital levels were included, the results might be representative of provinces or regions with similar socioeconomic characteristics, rather than the entire Vietnamese population. Fifth, the direct medical costs might have been overestimated because the majority of patients had secondary diagnoses or comorbid conditions. In such cases, total medical costs reflect the combined management of all conditions, not just the primary one, which can increase the estimated direct medical costs for the primary infectious condition. Sixth, this study included only public hospitals and did not capture cases treated in the private healthcare sector. As private facilities may differ in pricing structures, service availability, and patient socioeconomic profiles, the estimated costs may not fully reflect the overall economic burden of respiratory infections across all healthcare providers. Seventh, although this study used patient data collected after the end of the COVID-19 pandemic in Vietnam, the analysis did not account for potential lingering effects of the pandemic, such as changes in patient behavior or shifts in resource prioritization, which could influence the identification of cost drivers. For example, the shift to remote or telehealth services can increase costs for digital platforms and remote monitoring while reducing in-person service costs. On the other hand, patients may be admitted with more severe conditions, which can prolong hospital stays and increase inpatient costs. Therefore, these changes could influence overall costs and alter the coefficients of the identified cost drivers, such as length of stay in this case. Finally, the use of complete-case analysis, excluding observations with missing data in the GLM model, may introduce selection bias and limit the generalizability of the findings.

## Conclusion

Hospitalized adults with respiratory infections in Thai Nguyen, Vietnam incurred a median direct medical cost of US$174.9. Costs were substantially higher for lower respiratory tract infections and were influenced by hospital level, length of stay, and comorbidities. These findings highlight the need for preventive measures and efficient hospital resource allocation in Vietnam.

## Supporting information

S1 Table Extraction periods of datasets from the included hospitals. (DOCX)

S2 Table Direct medical costs of episodes by primary diagnosis (ICD-10 code) in US$. (DOCX)
