# Supplementary material for: Direct medical costs of respiratory infections in adults: A multicenter retrospective analysis in Thai Nguyen, Vietnam
Source: PLoS One. 2026 Jul 23;21(7):e0354461. doi: 10.1371/journal.pone.0354461 (PMC13395356; doi:10.1371/journal.pone.0354461)
Supplement: S1 Table — (DOCX) [file pone.0354461.s001.docx]

**SUPPLEMENTAL MATERIAL**

**S1. Extraction periods of datasets from the included hospitals**

| **No.** | **Hospital** | **Extraction period** | **Access date** |
| --- | --- | --- | --- |
| 1 | Thai Nguyen national hospital | October 2023 to December 2024 | 07 Oct 2025 |
| 2 | Gang Thep hospital | December 2022 to June 2025 | 19 Oct 2025 |
| 3 | Thai Nguyen lung hospital | March 2023 to May 2025 | 27 Oct 2025 |
| 4 | Thai Nguyen A hospital | December 2023 to March 2025 | 09 Oct 2025 |
| 5 | Phu Binh general hospital | December 2022 to June 2025 | 25 Oct 2025 |
| 6 | Dong Hy general hospital | January 2023 to December 2024 | 14 Oct 2025 |
| 7 | Vo Nhai general hospital | December 2022 to March 2025 | 10 Oct 2025 |
