# Supplementary material for: Direct medical costs of respiratory infections in adults: A multicenter retrospective analysis in Thai Nguyen, Vietnam
Source: PLoS One. 2026 Jul 23;21(7):e0354461. doi: 10.1371/journal.pone.0354461 (PMC13395356; doi:10.1371/journal.pone.0354461)
Supplement: S2 Table — (DOCX) [file pone.0354461.s002.docx]

**SUPPLEMENTAL MATERIAL**

**S2. Direct medical costs of episodes by primary diagnosis (ICD-10 code) in US$**

| **Variable** | **Overall**  n = 27,992 | **J00**  n = 65 | **J01**  n = 805 | **J02**  n = 1,452 | **J03**  n = 250 | **J04**  n = 302 | **J06**  n = 60 |
| --- | --- | --- | --- | --- | --- | --- | --- |
| Length of stay (days) | 9.4 (4.7) | 6.3 (4.0) | 5.3 (4.1) | 3.6 (3.9) | 5.9 (2.4) | 6.8 (2.9) | 6.1 (3.2) |
|  |  |  | **Total costs - Mean (Standard deviation)** | | |  |  |
| Total amount | 344.3 (1,478.7) | 182.7 (285.8) | 142.9 (256.9) | 101.7 (214.3) | 144.3 (225.8) | 148.4 (228.8) | 212.5 (273.8) |
| Covered by insurance | 294.0 (1,233.0) | 124.0 (138.1) | 112.7 (216.0) | 84.2 (180.9) | 112.7 (145.0) | 128.5 (197.7) | 181.8 (244.9) |
| Paid by patients | 45.8 (266.4) | 56.9 (159.9) | 29.4 (69.4) | 16.8 (58.8) | 30.7 (122.3) | 19.3 (47.1) | 27.2 (36.6) |
|  |  |  | **Cost components - Mean (Standard deviation)** | | |  |  |
| Laboratory tests | 40.0 (166.8) | 24.1 (107.9) | 13.4 (51.2) | 9.2 (33.1) | 15.0 (43.9) | 9.2 (24.9) | 20.2 (57.0) |
| Imaging diagnostics | 17.1 (63.6) | 12.5 (59.3) | 10.7 (28.7) | 5.0 (16.2) | 4.7 (18.2) | 3.9 (12.7) | 12.5 (32.9) |
| Medicines | 112.4 (553.1) | 56.6 (62.6) | 51.8 (86.9) | 37.7 (86.9) | 56.0 (65.5) | 58.5 (96.5) | 68.7 (80.0) |
| Blood products | 6.0 (98.4) | 0.0 (0.0) | 0.0 (0.0) | 0.1 (1.7) | 0.0 (0.0) | 0.0 (0.0) | 0.0 (0.0) |
| Procedures | 26.4 (279.9) | 8.4 (24.0) | 3.8 (15.8) | 3.8 (31.9) | 2.1 (12.3) | 4.5 (35.8) | 10.4 (50.4) |
| Medical supplies | 10.1 (192.8) | 3.4 (11.2) | 2.0 (6.0) | 1.4 (4.3) | 2.2 (6.1) | 1.8 (6.2) | 4.0 (9.2) |
| Consultation | 1.8 (2.3) | 1.6 (0.9) | 1.5 (1.9) | 1.5 (2.4) | 1.5 (2.4) | 1.4 (1.5) | 2.1 (1.4) |
| Hospital bed day | 129.1 (428.1) | 75.4 (99.3) | 59.1 (100.5) | 42.7 (82.0) | 62.4 (105.0) | 68.9 (83.9) | 91.7 (102.9) |
| Transportation | 0.2 (4.9) | 0.0 (0.0) | 0.2 (5.2) | 0.0 (0.0) | 0.0 (0.0) | 0.0 (0.0) | 0.0 (0.0) |
| Other services | 0.1 (5.3) | 0.0 (0.0) | 0.0 (0.0) | 0.0 (0.0) | 0.0 (0.0) | 0.0 (0.0) | 0.0 (0.0) |

*J00: Acute nasopharyngitis [common cold]; J01: Acute sinusitis; J02: Acute pharyngitis; J03: Acute tonsillitis; J04: Acute laryngitis and tracheitis; J06: Acute upper respiratory infections of multiple and unspecified sites.*

**S2.** **Direct medical costs of episodes by primary diagnosis (ICD-10 code) in US$ (Cont.)**

| **Variable** | **J09**  n = 553 | **J10**  n = 64 | **J11**  n = 187 | **J15**  n = 5,227 | **J16**  n = 51 | **J18**  n = 16,387 | **J20**  n = 2,285 | **J22**  n = 304 |
| --- | --- | --- | --- | --- | --- | --- | --- | --- |
| Length of stay (days) | 4.9 (2.5) | 6.2 (3.3) | 5.0 (2.5) | 11.8 (4.0) | 9.5 (6.9) | 10.1 (4.3) | 6.4 (3.8) | 8.7 (2.9) |
|  |  |  | **Total costs - Mean (Standard deviation)** | | |  |  |  |
| Total amount | 221.7 (586.3) | 1,128.6 (1,822.6) | 103.7 (82.6) | 439.0 (1,943.7) | 2,637.1 (3,422.9) | 378.3 (1,553.2) | 161.3 (267.4) | 194.1 (106.5) |
| Covered by insurance | 168.9 (445.8) | 915.1 (1,408.0) | 82.9 (63.2) | 376.0 (1,694.6) | 2,219.1 (2,923.1) | 325.6 (1,265.2) | 131.6 (198.1) | 162.6 (85.3) |
| Paid by patients | 50.9 (153.0) | 199.3 (450.7) | 20.8 (27.9) | 57.8 (251.0) | 359.1 (600.6) | 47.3 (309.6) | 28.8 (87.1) | 31.5 (37.0) |
|  |  |  | **Cost components – Mean (Standard deviation)** | | |  |  |  |
| Laboratory tests | 45.3 (137.6) | 181.1 (309.2) | 16.7 (16.9) | 55.9 (231.1) | 344.1 (472.8) | 41.7 (165.3) | 20.9 (59.2) | 19.5 (14.0) |
| Imaging diagnostics | 14.9 (47.0) | 56.9 (87.3) | 8.4 (6.7) | 19.4 (72.4) | 116.4 (160.4) | 19.0 (69.2) | 10.1 (25.8) | 11.2 (12.1) |
| Medicines | 41.7 (176.4) | 456.6 (1,176.4) | 18.3 (26.8) | 150.5 (817.3) | 892.9 (1,369.3) | 121.1 (539.1) | 52.9 (84.0) | 60.7 (46.8) |
| Blood products | 10.5 (163.6) | 0.0 (0.0) | 0.0 (0.0) | 9.4 (143.6) | 88.9 (361.6) | 6.5 (92.8) | 0.5 (13.3) | 0.2 (2.6) |
| Procedures | 3.3 (29.1) | 20.3 (53.6) | 0.5 (2.6) | 35.7 (334.0) | 329.5 (701.2) | 31.0 (309.7) | 4.7 (32.2) | 4.3 (11.5) |
| Medical supplies | 4.0 (9.2) | 23.4 (27.8) | 1.2 (1.2) | 12.8 (190.4) | 74.6 (125.1) | 12.1 (227.7) | 2.3 (6.1) | 2.4 (1.9) |
| Consultation | 2.8 (3.1) | 7.2 (5.2) | 1.9 (1.4) | 1.6 (1.8) | 5.9 (6.1) | 1.8 (2.4) | 1.8 (2.1) | 2.0 (0.8) |
| Hospital bed day | 98.0 (134.4) | 379.0 (434.8) | 55.4 (50.4) | 151.9 (460.7) | 770.4 (1,008.1) | 143.7 (484.0) | 67.6 (105.4) | 90.6 (50.9) |
| Transportation | 0.0 (0.0) | 0.0 (0.0) | 0.0 (0.0) | 0.8 (9.9) | 0.0 (0.0) | 0.2 (3.8) | 0.2 (6.3) | 1.3 (10.7) |
| Other services | 0.0 (0.0) | 0.0 (0.0) | 0.0 (0.0) | 0.1 (4.3) | 0.9 (6.6) | 0.1 (6.5) | 0.0 (0.0) | 0.0 (0.0) |

*J09: Influenza due to certain identified influenza virus; J10: Influenza due to identified seasonal influenza virus; J11: Influenza, virus not identified; IQR: Interquartile range; J15: Bacterial pneumonia, not elsewhere classified; J16: Pneumonia due to other infectious organisms, not elsewhere classified; J18: Pneumonia, organism unspecified; J20: Acute bronchitis; J22: Unspecified acute lower respiratory infection*
